# MiR-760 exerts a critical regulatory role in glioma proliferation, migration, and invasion by modulating MMP2 expression

**DOI:** 10.7150/jca.92518

**Published:** 2024-04-08

**Authors:** Zhengting Qian, Heng Xin, Zhen Jia, Jiageng Xia, Yong Tang, Xiang Li, Heming Wu, Youwu Fan

**Affiliations:** 1 Nanjing Medical University, 210000, Nanjing, JiangSu, China.; 2 Department of Neurosurgery, Nanjing First Hospital, Nanjing Medical University, Nanjing, Jiangsu, 210006, China.

**Keywords:** miR-760, MMP2, proliferation, migration, invasion, glioma

## Abstract

**Background:** Glioma represents the predominant subtype of brain tumor, characterized by an unfavorable prognosis. Current evidence indicates the involvement of microRNAs (miRNAs) in the initiation and progression of glioma malignancies. While miR-760 has been recognized in the context of tumorigenesis, its precise role in gliomas remains insufficiently explored.

**Methods:** In this investigation, we harnessed the GSE25631 database to scrutinize the aberrant expression profiles of microRNAs, whereby the diminished expression of miR-760 in glioblastoma was validated. Our aim was to delineate the expression patterns of microRNA-760 (miR-760) and probe its prognostic significance within the realm of glioma. Employing quantitative real-time polymerase chain reaction, we ascertained the relative expression levels of miR-760 and MMP2 in glioma cell lines. The impact of miR-760 on cell proliferation, migration, and invasion was assessed through Cell Counting Kit-8 (CCK-8), 5-ethynyl-2'-deoxyuridine (EdU), and Transwell assays. Bioinformatics analysis corroborated the downstream target gene of miR-760. Furthermore, a luciferase reporter experiment was conducted to pinpoint MMP2 as the direct target gene of miR-760. The assessment of MMP2 protein levels was accomplished through Western blotting and immunofluorescence techniques.

**Result:** Our data unequivocally revealed a substantial reduction in miR-760 expression within glioma tissues and cell lines. Heightened miR-760 levels exerted a restraining influence on the proliferation, migration, and invasion capabilities of glioma cell lines. The outcomes of our bioinformatics analysis unveiled the ability of miR-760 to engage with and curtail MMP2 expression. Collectively, these findings posit that miR-760 exerts a restraining influence on glioma growth by orchestrating the upregulation of miR-760 along the miR-760/MMP2 axis.

**Conclusion:** The delineation of the miR-760/MMP2 axis promises to broaden our comprehension of the intricate molecular mechanisms underpinning glioma proliferation and may unveil prospective therapeutic avenues for the management of glioma.

## Introduction

Histologically, gliomas are classified into four distinct histological subtypes according to the grading system of the World Health Organization (WHO) 1. Glioblastoma multiforme (GBM) is the most prevalent kind of primary brain cancer in adults, and there are currently no effective treatments available 2, 3. Patients with glioblastoma have an exceedingly poor clinical prognosis, with a median survival of approximately 1.5 years 4-6. Thus, understanding these molecular pathways is critical for improving the prognosis and responsiveness to therapy of glioma patients.

MicroRNAs (miRNAs) have been identified as unique types of potential diagnostic biomarkers and therapeutic targets for a variety of human disorders, including tumours 7-9. miRNAs are extremely conserved, small noncoding RNA molecules that may bind to the 3′-untranslated region (3′-UTR) of a target gene mRNA through base matching pairing and modulate the expression levels of the mRNA by blocking its translation or degrading it 10, 11. Numerous miRNAs have been shown to be abnormally expressed in a variety of malignancies, indicating that miRNAs may act as tumour suppressors or oncogenes 12-14.

miR-25 contribute to lung cancer cell proliferation and metastasis by targeting the LATS2/YAP signalling pathway 12. Furthermore, overexpression of miRNA-338-3p suppressed cell proliferation, migration, invasion, and promoted apoptosis of glioma by targeting MYT1L 15. The absence of evidence for the participation of miR-760 in glioma motivated us to examine its biological activities and underlying mechanism.

In the current investigation, miR-760 was considerably downregulated in glioma tissues compared to normal tissues, according to our bioinformatics investigation. Bioinformatics analysis and a double luciferase reporter gene experiment were used to identify MMP2 mRNA as a microRNA downstream target. The expression levels of miRNAs were investigated by reverse transcription-quantitative polymerase chain reaction (qRT‒PCR) in glioma cell lines and normal human astrocytes (NHAs). The effects of miR-760 on glioma cell line proliferation, migration, and invasion *in vitro* were investigated at the molecular level. We investigated the molecular mechanism and underlying processes by which miR-760 functions in glioma cell lines *in vitro*. Additionally, we investigated the molecular mechanism by which miR-760 influences glioma cell proliferation, migration, and invasion through MMP2 regulation.

## Materials and methods

### Cell Lines

Glioma cells (U87, U251, T98, U118) were obtained from Procell (Wuhan, China), and the cells were grown in Dulbecco's modified Eagle's medium (DMEM) (Gibco Company, Grand Island, NY, USA) supplemented with 10% foetal bovine serum (FBS) (Gibco Company, Grand Island, NY, USA). Normal human astrocytes (NHAs), which were grown in astrocyte media (AM, ScienCell, USA, Cat. No. 1801), were provided by Sciencell Research Laboratories. Cell lines were cultivated at 37 °C with 5% CO_2_ in a humid incubator.

### Microarray data analysis

The GSE25631 dataset includes sequencing data from the GEO database. The GSE25631 dataset includes 82 primary glioblastoma multiforme surgical specimens and 5 normal brain tissues from locations around arteriovenous malformations.

### Quantitative real‑time polymerase chain reaction (qRT‑PCR)

An RNA isolater (Vazyme, Nanjing, China) was used to extract total RNA from cell lines, as directed by the manufacturer. According to the manufacturer's instructions, cDNA was produced using a miRNA 1st Strand cDNA Synthesis Kit (by stem‒loop) (Vazyme, Nanjing, China). Vazyme created stem loop miRNA qRT‒PCR primers specific for miR-760 and U6. (Generay, Shanghai, China). The primers mentioned in this paper are shown in Table 1. The CT results were analysed by the 2^-ΔΔCT^ method.

### Cell Transfection

MiR-760 and NC mimics, shRNA for MMP2 knockdown (sh-MMP2) and the negative control (sh-NC), and a construct for MMP2 overexpression and the control were manufactured at GenePharma Company (Shanghai, China). The sequences mentioned in this article are shown in Table 2. Lipofectamine 3000 (Invitrogen) was utilized to transfect cells according to the manufacturer's recommendations. The relative expression of miR-760 and MMP2 was measured 48 hours later using a qRT‒PCR experiment.

### Dual-luciferase reporter assay

The wild-type (WT) and mutant-type (MUT) 3′UTR (nontranslated region) sequences of MMP2, including the miR-760 binding site, were first constructed. These sequences were created by GeneChem (GeneChem Co., Ltd. Shanghai, China). miR-760 mimics and inhibitors were transfected into glioma cells using Lipofectamine 3000, as well as MMP2-WT or MMP2-MUT. The cells were then grown for an additional 48 hours before being harvested. The Dual-Luciferase Reporter Assay System was then used to calculate the luciferase activity (Promega, USA). Experiments were conducted according to the manufacturers' instructions.

### EdU assay

The KeyFluor555 Click-iT EdU Kit (Keygen, Jiangsu, China) was used for the 5-ethynyl-2'-deoxyuridine (EdU) test. Transfected U87 and U251 cells were plated at a density of 2×10^4^ cells per well in 24-well culture plates and cultivated for 12 hours. After an additional 12 hours of incubation, PBS was used to rinse the treated cells twice. The cells were fixed with 4% paraformaldehyde for minutes at 37 °C after being incubated with 10 μM EdU solution at 37 °C for 2 hours. For 30 minutes, the nuclei of the cells were stained with DAPI. Finally, using a fluorescence microscope (Carl Zeiss, Germany), EdU-positive cells were observed and assessed. The proliferation ratio was determined by counting EdU-positive cells and then dividing the number of EdU-positive cells by the total number of cells.

### Transwell assay

U87 and U251 cells were cultured at 37 °C for 24 hours in the top chamber with or without Matrigel after transfection pretreatment (BD Biosciences). After 48 hours, the cells that migrated to the bottom side of the membrane were stained with crystal violet (Beyotime), and the cells that remained on the to surface of the membrane were removed using a cotton swab.

### CCK-8 assay

Cells were seeded and cultured in 96-well plates at density of 2000 cells per well. At 0, 24, 48, and 72 hours, CCK8 working solutions were added, and the culture continued for an extra 2 hours. The wavelength was measured at each 450 nm time point.

### Western blotting

To extract total protein, cells were lysed according to the manufacturer's instructions using radioimmunoprecipitation assay (RIPA) lysis buffer (Yifence Bio TECH, Nanjing, China).

SDS‒PAGE was used to separate the proteins, which were then transferred to polyvinylidene difluoride (PVDF) membranes (Millipore Corporation, Billerica, USA). After one hour of blocking with 5% skim milk, the membranes were incubated at 4 °C for 12 hours with primary antibodies (anti-MMP2 and anti-β-actin). Then, a secondary antibody conjugated to HRP was added and incubated for an additional 1 hour. To measure protein expression, an enhanced chemiluminescent system (Biosharp, Hefei, China) was utilized. In this experiment, β-actin was employed as a reference gene.

### Immunofluorescence staining

U87 and U251 cells were grown on glass coverslips until they were 50% confluent and then fixed for 30 minutes with 4% paraformaldehyde. After that, the cells were blocked with donkey serum for minutes at 37 °C and then coated overnight at 4 °C with anti-MMP2 antibody (1:50, MMP2, Proteintech, China), followed by 1 hour at 37 °C with goat anti-rabbit IgG (1:200, Proteintech, China). The nuclei were then stained for 5 minutes with 4′,6-diamidino-2-phenylindole (DAPI; Beyotime, China). Finally, the cells were examined using fluorescence microscopy.

### Statistical Analysis

SPSS 20.0 (IBM Corp, Armonk, NY, USA) was used to process the data. The data are presented as the mean±SD of three separate experiments. Analysis of variances was applied using Student's t test and one-way ANOVA, and P<0.05 was deemed statistically significant.

## Result

### The expression of miR-760 decreased in glioma

**Figure 1:**
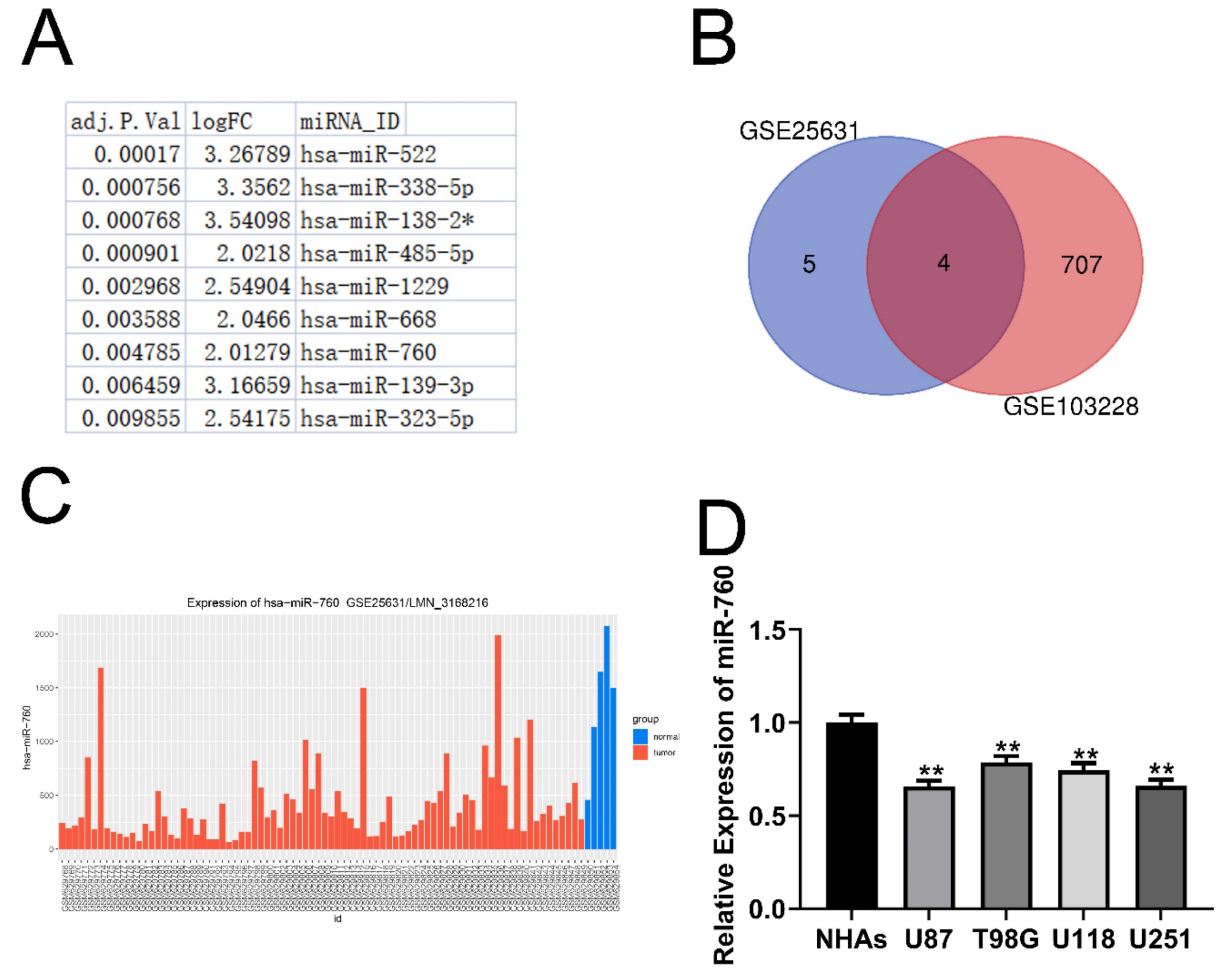
**miR-760 expression is decreased in glioma tissues and cell lines. (A**. Eleven miRNAs that were expressed at significantly low levels in glioma were identified in the GSE25631 database. **(B)** Four miRNAs were obtained after the intersection of GSE25631 and GSE103228. **(C)** The expression of miR-760 was measured in normal and glioma tissues from the GSE25631 database. **(D)** The expression of miR-760 was measured in different glioma cell lines and normal human astrocytes.

In the GSE25631 dataset, we discovered 11 miRNAs that were substantially differentially expressed in glioma (Fig. 1A adjusted P<0.05, logFC>=2). Furthermore, 4 miRNAs were identified after intersecting the miRNAs mentioned above with the miRNAs that were expressed at low levels in GSE103228 (Fig. 1B). This suggests that the expression of these 4 miRNAs was decreased in glioma tissues compared to normal brain tissues. Through the analysis of the GSE25631 database, we identified miR-760 as our research object (Fig. 1C). Moreover, glioma cell lines expressed considerably less miR-760 than NHAs (Fig. 1D All P<0.01). MiR-760 has been shown to inhibit the proliferation and metastasis of colorectal cancer, non-small cell lung cancer and liver cancer 16-18. However, few studies have evaluated whether miR-760 has a function in glioma. The molecular mechanism of miR-760 is still unknown.

### MMP2 is a Target of miR-760

**Figure 2:**
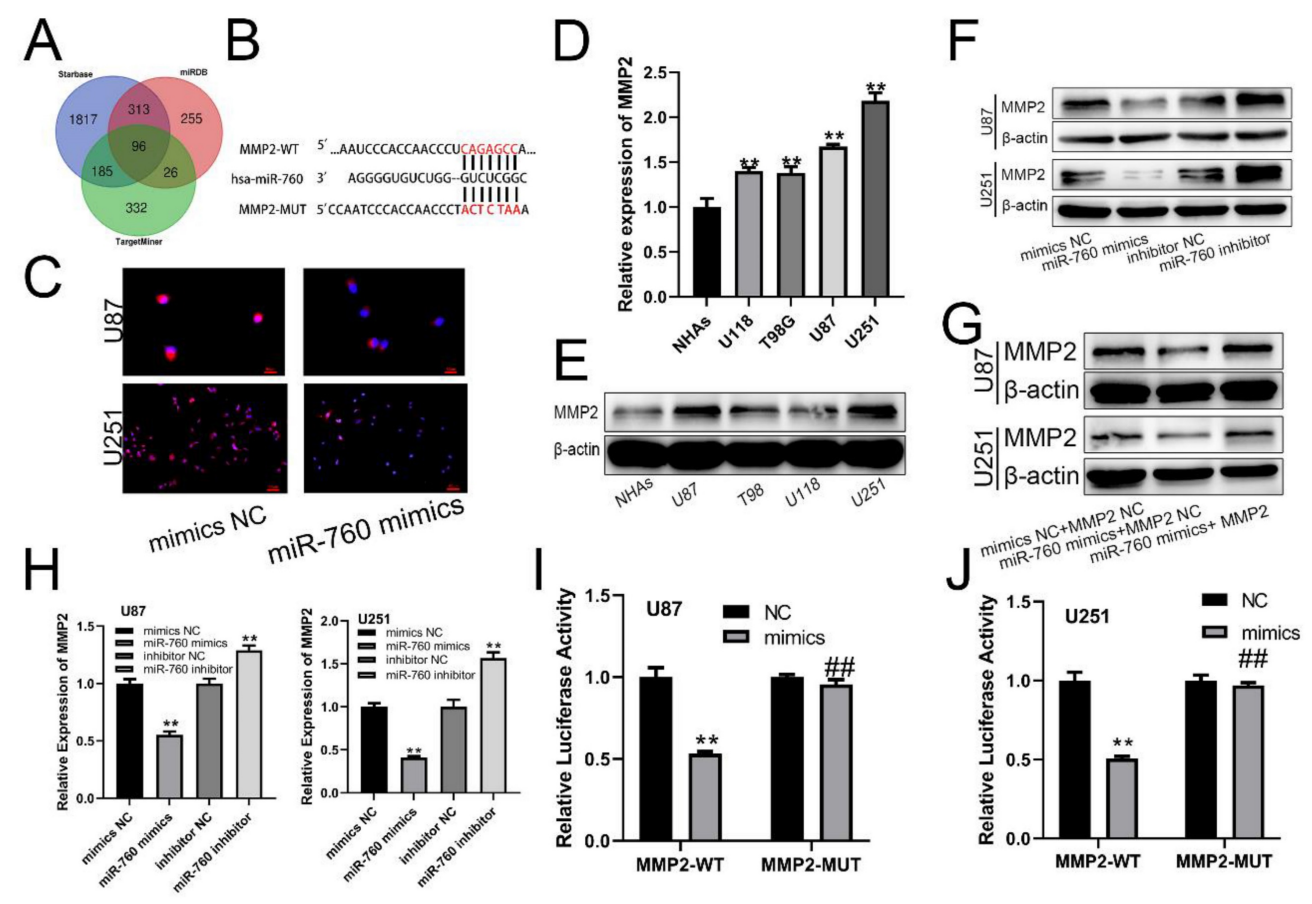
**miR-760 binds to MMP2 and regulates its expression. (A)** Analyses of the starBase, miRDB, and TargetMiner databases all identified the miR-760 target gene. **(B)** Wild-type and Mut-type sequences of the MMP2 3'UTR, which contained binding sites for miR-760. **(C)** Immunofluorescence analysis of MMP2 in U87 and U251 cell lines transfected with the inhibitors NC and miR-760. **(D)** qRT‒PCR and Western blotting analysis identified a difference in MMP2 expression between glioma and normal human astrocyte cell lines. **(E)** MMP2 expression in different groups of cell lines as shown by Western blotting analysis. **(F)** The expression of MMP2 in U87 and U251 cell lines transfected with miR-760 mimics, miR-760 inhibitor and the corresponding controls as shown by Western blotting. **(G)** The expression of MMP2 in U87 and U251 cell lines transfected with miR-760 mimics, MMP2 overexpression plasmid and the corresponding controls as shown by Western blotting. **(H)** The relative expression of MMP2 in the mimic NC, miR-760 mimic, inhibitor NC and miR-760 inhibitor groups in U87 and U251 cell lines measured by qRT‒PCR. (*). The relative luciferase activity of MMP2-WT and MMP2-MUT groups after the U87 and U251 cell lines were transfected with NC and miR-760 mimics, respectively. The data are expressed as the mean ± SD, **P < 0.01.

The miRDB, starBase, and TargetMiner databases all revealed the indicated mRNA as a miR-760 target gene (Fig. 2A). There was a binding sequence between MMP2 and miR-760 (Fig. 2B). Similarly, MMP2 expression was similarly suppressed by miR-760-mimics according to immunofluorescence (Fig. 2C). Notably, increased MMP2 expression levels were identified in glioma cell lines using qRT‒PCR analysis (Fig. 2D All p<0.01) and Western blotting assay (Fig. 2E). miR-760 mimics and an inhibitor of miR-760 were transfected into U87 and U251 cells, and the MMP2 protein expression level was determined using Western blotting. The level of MMP2 in the miR-760 mimics+MMP2 NC transfection group was downregulated compared with that in the mimics NC+MMP2 NC group, and this downregulation effect could be recovered by the MMP2 overexpression plasmid (Fig. 2G). The findings indicated that the level of MMP2 expression was lower in the miR-760 mimic transfection group than in the mimic NC group, but it was increased in the miR-760 inhibitor group compared with the inhibitor NC group, as shown by qRT‒PCR (Fig. 2H) and Western blotting assays. As shown by the luciferase reporter gene test, miR-760 mimic transfection in the MMP2-WT group decreased luciferase activity compared to that in the NC group. However, miR-760 transfection in the MMP2-MUT group had almost no effect on double luciferase activity (Fig. 2I, J). The data above demonstrate that miR-760 specifically targets and controls MMP2 expression.

### miR-760 regulates glioma cell proliferation, migration and invasion by targeting MMP2

**Figure 3:**
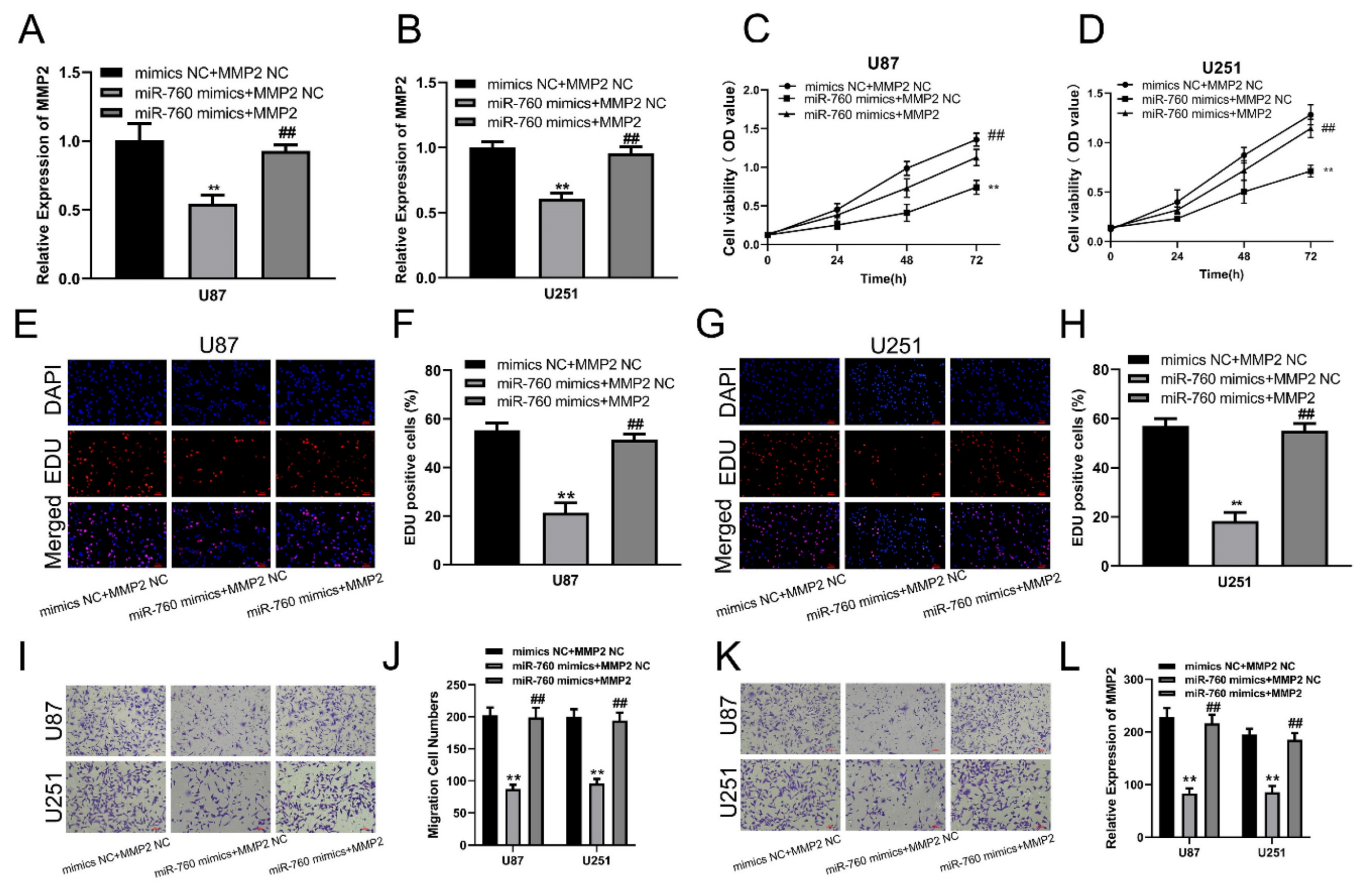
** The effect of miR-760 on gliomas is partially mediated by MMP2. (A, B)** The relative expression of MMP2 in U87 and U251 groups transfected with miR-760 mimics, MMP2 overexpressed plasmid groups or the corresponding controls tested by qRT‒PCR. **(C, D)** The CCK-8 assay was used to test cell viability in U87 and U251 cell lines transfected with miR-760 mimic, MMP2-overexpressing groups or the corresponding controls. **(E-H)** The EdU assay was used to test U87 and U251 cells transfected with miR-760 mimics, MMP2 overexpression plasmid groups or the corresponding controls. **(I-J)** The migration of U87 and U251 cells transfected with miR-760 mimics, MMP2 overexpression plasmid groups or the corresponding controls was detected and analysed statistically. **(K-L)** The invasion of U87 and U251 cells transfected with miR-760 mimics, MMP2 overexpression plasmid groups or the corresponding controls was assessed and analysed statistically. The data are expressed as the mean ± SD, **P < 0.01

qRT‒PCR was used to measure the expression level of MMP2 after transfection of miR-760 mimics and cotransfection of miR-760 mimics and MMP2 expression plasmid. The results showed that the MMP2 expression level was significantly downregulated after transfection with miR-760 mimics, and this effect could be reversed by the MMP2 overexpression plasmid (Fig. 3A, B, p<0.01). CCK-8 assays were used to determine the proliferative capacity of miR-760. The CCK-8 experiment indicated that upregulating miR-760 had a negative effect on the capacity of glioma cells to grow; conversely, this effect could be restored by the MMP2 overexpression plasmid on cell proliferation (Fig. 3C, D, p<0.01). The EdU assay produced comparable results to the CCK-8 assay, indicating that miR-760 overexpression decreased glioma cell growth and that this effect could be reversed by MMP2 overexpression plasmid transfection (Fig. 3E-H, p<0.01). Transwell assays were then used to determine the effect of miR-760 on the migratory and invasive capabilities of U87 and U251 cells. Transwell studies indicated that miR-760 mimics significantly inhibited the migration and invasion of U87 and U251 cells, and this effect could be reversed by MMP2 overexpression plasmid transfection. (Fig. 3I-L, p<0.01).

### MMP2 downregulation can inhibit the viability and proliferation of glioma cells

**Figure 4:**
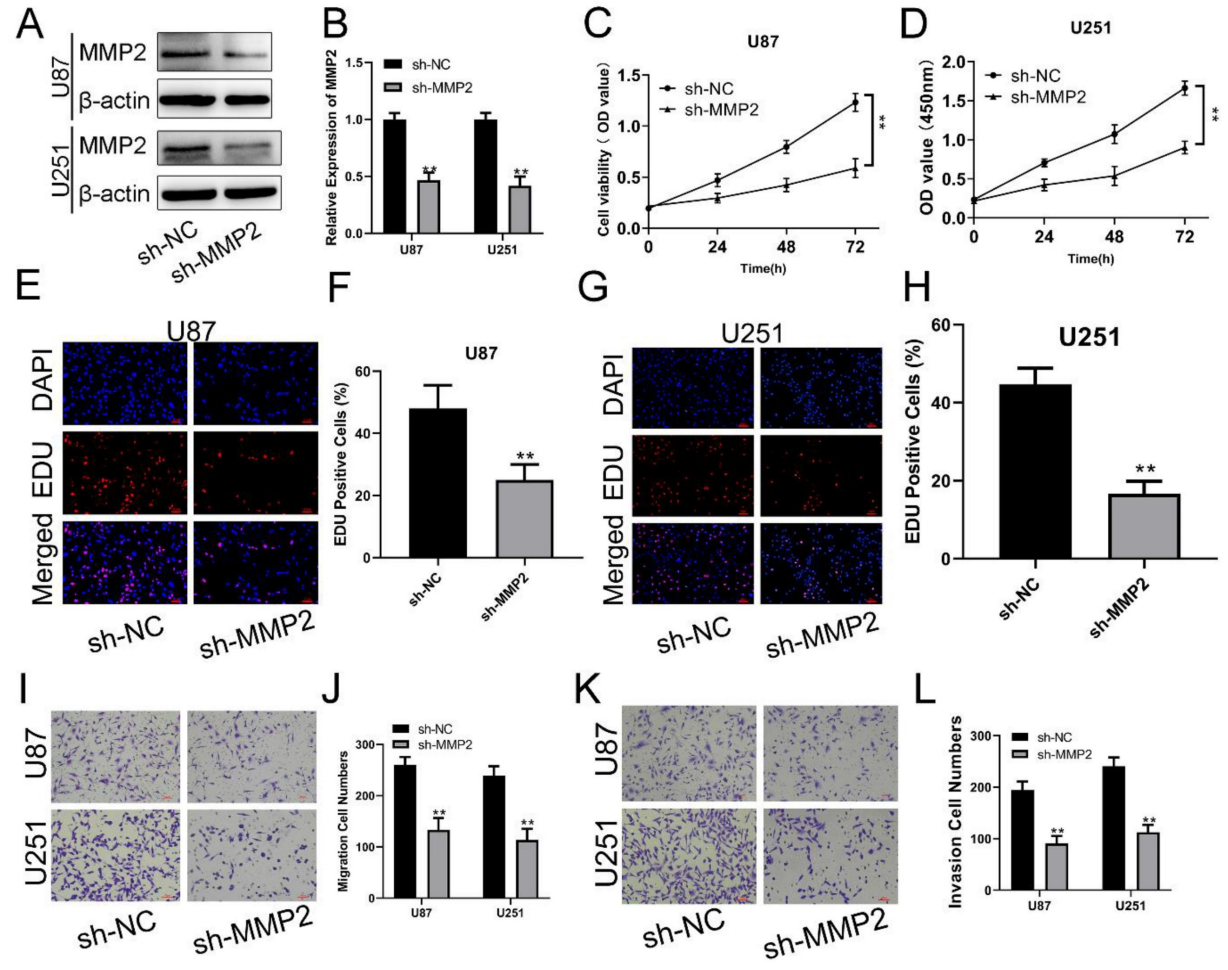
** MMP2 affects the proliferation, migration and invasion of glioma. (A)** The relative expression of MMP2 in sh-NC and sh-MMP2 groups tested by Western blotting in U87 and U251 groups. **(B)** The relative expression of MMP2 in sh-NC and sh-MMP2 groups tested by qRT‒PCR in U87 and U251 groups. **(C)** The CCK-8 assay was used to test cell viability in the U87 and U251 cells in the sh-NC and sh-MMP2 groups. **(E-H)** The EdU assay was used to test U87 and U251 cells transfected with sh-NC and sh-MMP2. **(I-J)** The migration of U87 and U251 cells transfected with sh-NC and sh-MMP2 was assessed and analysed statistically. **(K-L)** The invasion of U87 and U251 cells transfected with sh-NC and sh-MMP2 was assessed and analysed statistically. The data are expressed as the mean ± SD, **P < 0.01

To examine the biological function of MMP2 in glioma, sh-NC and sh-MMP2 were transfected into U87 and U251 cells, respectively. The effectiveness of transfection was determined using qRT‒PCR and Western blotting assays (Fig. 4A, B). CCK-8 and EdU assays were used to determine the proliferation of U87MG and U251 cells. MMP2 silencing dramatically decreased the viability of U87 and U251 cells (Fig. 4C, D). The EdU test was used to determine the involvement of MMP2 in U87 and U251 cell growth. As demonstrated in Fig. 4E-H (p<0.01), sh-MMP2-transfected U87 and U251 cells drastically decreased EDU-positive cells when compared to the sh-NC group (Fig. E-H). The Transwell results also showed that the sh-MMP2 group significantly inhibited the migration and invasion of U87 and U251 cells compared with the sh-NC group (Fig. I-L p<0.01). These results indicate that downregulation of MMP2 can significantly inhibit the proliferation, migration and invasion of glioma cells.

## Discussion

Glioma is the most prevalent kind of cancer of the nervous system 19, 20. Glioma, particularly high-grade glioma, has a poor prognosis 21. Currently available glioma treatments have shown minimal efficacy in terms of patient outcome, emphasizing the necessity of novel molecularly targeted therapeutics 22-26. At present, it is becoming more obvious that miRNAs are involved in almost all cell activities associated with human malignancy and play a significant role in cancer formation 27-29. Moreover, cancer-associated altered miRNA expression patterns may serve as prognostic and/or diagnostic indicators 30-32. Changes in miRNA expression have indeed been implicated in the aetiology of glioma.

MiR-760 has been reported to suppress carcinogenesis and tumor progression in many human tumors. It has been shown that miR-760 was found to reduce the cancer stem cell population and inhibit breast cancer cell proliferation and metastasis via inactivation of NANOG transcription factor 33. However, the clinical relevance and biological role of miR-760 in glioma remain unclear. The present study demonstrated that miR-760 expression was considerably lower in glioma cell lines than in normal human astrocytes. The upregulation of miR-760 expression greatly inhibited glioma cell proliferation, migration, and invasion. A bioinformatic analysis tool and a dual-luciferase activity reporter experiment were used to identify MMP2 as a potential target of miR-760 in glioma cells. MMP2 pertains to the zinc-dependent endopeptidases family, adept at degrading components of the extracellular matrix (ECM). It plays pivotal roles in tumor cell growth, differentiation, invasion, metastasis, modulation of tumor angiogenesis, and immune surveillance 34. The expression of MMP2 has been associated with glioma invasion. Inhibiting AKT using the inhibitor API-1 decreased MMP2 expression, suggesting that CK1 controlled MMP2 via the AKT pathway, enhancing glioma cell invasion 35. Additionally, MMP2 expression was greatly increased in glioma samples and was negatively correlated with miR-760 expression. Moreover, upregulation of miR-760 expression suppressed MMP2 expression in glioma cells, while MMP2 upregulation enhanced glioma cell proliferation, migration, and invasion.

In conclusion, the GSE25631 database showed that the expression of miR-760 was downregulated in glioma tissues and cell lines. The current work has shown that miR-760 is downregulated in glioma cell lines compared to normal human astrocytes, and miR-760 exerts a tumour suppressive effect in glioma by decreasing cell proliferation and invasion by directly targeting MMP2, suggesting that miR-760 might be a new predictive biomarker and a potential therapeutic target for glioma. However, due to the current limitations in experimental conditions, we have opted for classical 2D cell lines, which often significantly impact phenotypes, cellular signalling, and drug responses. In subsequent experiments, we contemplate the utilization of glioma stem cells (GCS) as a further option. In addition, more *in vivo* research is required to confirm the tumour-suppressing role of miR-760 in glioma.

## Figures and Tables

**Table 1:** The primers used in this study.

Primer	Forward	Reverse
has-miR-760	CGGGCCGGCTCTGGGTCT	CAGCCACAAAAGAGCACAAT
MMP2	GATACCCCTTTGACGGTAAGGA	CCTTCTCCCAAGGTCCATAGC
GAPDH	CATGAGAAGTATGACAACAGCCT	AGTCCTTCCACGATACCAAAGT
U6	CAGCACATATACTAAAATTGGAACG	ACGAATTTGCGTGTCATCC

**Table 2:** The interfering nucleotide used in this study.

	Sequence	Sequence
Plasmid sequences	Sense (5'-3')	Antisense (5'-3')
hsa-miR-760 mimics	CGGCUCUGGGUCUGUGGGGA	CCCACAGACCCAGAGCCGUU
hsa-miR-760 inhibitor	UCCCCACAGACCCAGAGCCG	
inhibitor NC	CAGUACUUUUGUGUAGUACAA	
sh-MMP2	GTTCTCCGAACGTGTCACGT	
sh-NC	GTTCTCCGAACGTGTCACGT	
NC	UUCUCCGAACGUGUCACGUTT	ACGUGACACGUUCGGAGAATT
